# A UK Specification for Trusted Research Environments

**DOI:** 10.23889/ijpds.v9i5.2609

**Published:** 2024-09-18

**Authors:** Hari Sood, Simon Li, Tim Machin, Katie Oldfield, Antony Chuter, Jillian Beggs, Sonya Coleman, Dermot Kerr, Ed Chalstrey, Matthew Craddock, Jim Madge, David Sarmiento-Perez, James Robinson, Cian O'Donovan, Martin O'Reilly, Christian Cole

**Affiliations:** 1 The Alan Turing Institute; 2 University of Dundee; 3 University College London; 4 Research Data Scotland; 5 Public Member; 6 Ulster University

## Objectives

The need for Trusted Research Environments (TREs) is clear. Several influential reports have highlighted that personal or sensitive data which have been collected for operational, commercial or governmental reasons need to be managed securely in an environment that encourages best practices.

TREs are designed to enable only authorised projects and researchers access to sensitive data whilst minimising risk of data exposure. Yet the TRE landscape has grown organically over at least the last decade resulting in heterogeneous environments, making  it harder for data to be discovered, shared and used for public benefit.

A baseline specification for TREs is required.

## Approach

We engaged with around 60 organisations covering government, industry, health and academia through regular, online, shared spaces or “Collaboration Cafés”. We also embedded the public voice within the project by having members of the public within the research team and developed a series of workshops to include and reflect their input to the architecture. The whole project was developed in the open to instil trustworthiness.

## Results

The SATRE project has produced a reference TRE architecture and implementation through significant engagement with the UK TRE community. This baseline specification for UK TREs includes a set of four pillars, 28 capabilities and 160 statements.

## Conclusions and Implications

An open and transparent TRE specification and architecture for a wide range of stakeholders has been developed. TREs can now use the SATRE specification to evaluate their environments against a common baseline.

